# *Plasmodium* infection reduces the volume of the viral reservoir in SIV-infected rhesus macaques receiving antiretroviral therapy

**DOI:** 10.1186/s12977-014-0112-x

**Published:** 2014-12-09

**Authors:** Xiao-Yong Zhan, Nina Wang, Guangjie Liu, Limei Qin, Wanwan Xu, Siting Zhao, Li Qin, Xiaoping Chen

**Affiliations:** Laboratory of Pathogen Biology, State Key Laboratory of Respiratory Disease, Center for Infection and Immunity, Guangzhou Institutes of Biomedicine and Health, Chinese Academy of Sciences, No. 190 Kaiyuan Avenue, Luogang District, Guangzhou Science Park, Guangzhou, 510530 Guangdong Province China

**Keywords:** HIV-1, SIV, AIDS, *Plasmodium*, Malaria, Rhesus macaque model, Co-infection, ART, Viral reservoir

## Abstract

**Background:**

Previous studies indicated that *Plasmodium* infection activates the immune system, including memory CD4+ T cells, which constitute the reservoir of human immunodeficiency virus type-1 (HIV-1). Therefore, we postulated that co-infection with malaria might activate the reservoir of HIV-1. To test this hypothesis, we used a rhesus macaque model of co-infection with malaria and simian immunodeficiency virus (SIV), along with antiretroviral therapy (ART).

**Results:**

Our results showed that *Plasmodium* infection reduced both the replication-competent virus pool in resting CD4+ T cells and the integrated virus DNA (iDNA) load in peripheral blood mononuclear cells in the monkeys. This reduction might be attributable to malaria-mediated activation and apoptotic induction of memory CD4+ T cells. Further studies indicated that histone acetylation and NF-kappaB (NF-κB) activation in resting CD4+ T cells may also play an important role in this reduction.

**Conclusions:**

The findings of this work expand our knowledge of the interaction between these two diseases. As more HIV-1-infected individuals in malaria-endemic areas receive ART, we should explore whether any of the patients co-infected with *Plasmodium* experience virologic benefits.

**Electronic supplementary material:**

The online version of this article (doi:10.1186/s12977-014-0112-x) contains supplementary material, which is available to authorized users.

## Background

Human immunodeficiency virus type-1 (HIV-1) infection is one of the most important global health problems [1]. The virus replicates within and destroys CD4+ T cells in patients, ultimately leading to acquired immune deficiency syndrome (AIDS). Antiretroviral therapy (ART) can dramatically control the replication of HIV-1 and decrease viral loads in HIV-1-infected individuals. Many HIV-1-positive individuals have recently begun receiving ART in low- and middle-income regions. The pool of resting CD4+ T cells, including CD4+ central memory T (T_CM_) and effector memory T (T_EM_) cells, is a major reservoir because ART does not affect the provirus within these cells [2,3]. These cells thus provide a long-lasting cellular reservoir for HIV-1 [2,3]. As a result, one of the major therapeutic strategies for eradication of the HIV-1 reservoir is to reactivate the latent provirus in resting CD4+ T cells [4]. This strategy is promising for purging HIV-1 from this reservoir.

Malaria is another important infectious disease, and it has been shown to strongly activate the immune system [5]. In particular, *Plasmodium* antigens can activate T cells via antigen-presenting cells (APCs) [5], and polyclonal activated T and B cells, including memory CD4+ T cells, are observed during the blood stage of *Plasmodium* infection in mice and monkeys [6-8].

Previous studies have suggested that malaria has effects on HIV-1 infection [9-12]. Specifically, malaria infection might strongly activate CD4+ T cells; up-regulate proinflammatory cytokines, such as interleukin-6 (IL-6) and tumor necrosis factor-alpha (TNF-α); and induce activation of the virus [9,13,14]. However, ART may control HIV-1 replication in CD4+ T cells, even during malaria infection. We therefore hypothesized that the impact of malaria on HIV-1 infection under ART might be different from the impact in those situations without ART. More specifically, we hypothesized that malaria infection might activate latently infected resting CD4+ T cells and induce latent virus reactivation, thus reducing the volume of the viral reservoir under ART.

To test our hypothesis, we utilized a rhesus macaque model of co-infection with *Plasmodium cynomolgi* (Pc, a nonlethal monkey malaria *Plasmodium* species) and simian immunodeficiency virus (SIVmac251, SIV) under ART. As expected, malaria activated the CD4+ T cells, decreased the integrated virus DNA (iDNA) load in peripheral blood mononuclear cells (PBMCs) and reduced the replication-competent virus pool in resting CD4+ T cells. We also found increased levels of apoptotic memory CD4+ T cells during the course of Pc infection. Additionally, malaria induced histone acetylation and activation of NF-kappaB (NF-κB) in resting CD4+ T cells. All of these factors might contribute to the reduction of the viral reservoir.

## Results

### Pc infection reduced the size of the viral reservoir in SIV-infected macaques

**Figure 1:**
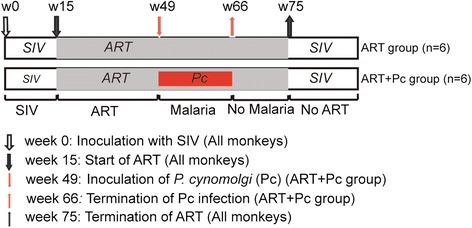
**Animal study design.** At week 0, 12 monkeys in the 2 groups were infected with SIV. At week 15, all monkeys started to receive ART (downward black arrow). During weeks 49–66, 6 monkeys in the ART + Pc group were co-infected with Pc (red arrows and box)*.* At week 75, ART was terminated in all monkeys (upward black arrow). The light gray boxes indicate ART, and the light red box indicates Pc infection.

The design of the animal experiments is shown in Figure 1. Twelve Chinese-origin rhesus macaques (*Macaca mulatta*) were inoculated intravenously with 10,000 50% tissue culture infectious doses of *SIVmac251* and were randomly divided into two groups (n = 6 per group): the ART group (ART only) and the ART + Pc group (ART plus Pc infection). SIV infection resulted in certain typical virological and immunological changes in the monkeys that were similar to those reported previously (Additional file 1: Figure S1) [15]. The animals in the ART + Pc group displayed typical clinical manifestations of malaria during Pc infection (data not shown).

**Figure 2:**
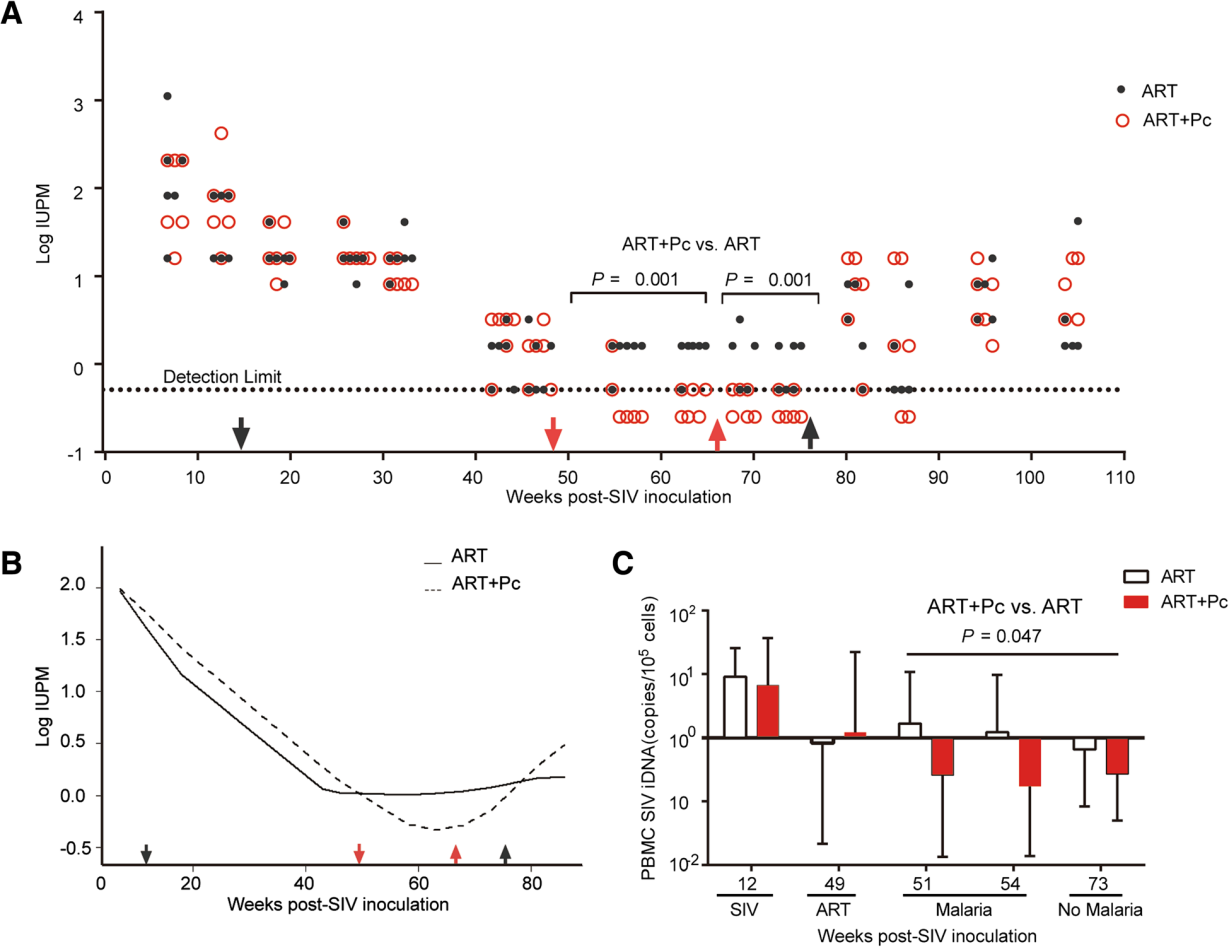
**The impact of Pc infection on the SIV reservoir. (A)** A significantly reduced frequency of resting CD4+ T cells harboring replication-competent SIV was observed during malaria infection in the ART + Pc group. Four of 6 monkeys in the ART + Pc group maintained less than 0.51 IUPM during the malaria phase, whereas all 6 monkeys in the ART group maintained an IUPM value above or equal to 0.51. The IUPM values in the two groups of monkeys were significantly different during the malaria phase. **(B)** The fitted curve revealed dynamic changes in IUPM in the two groups during the study. Each arrow has the same meaning as indicated in Figure 1. **(C)** Dynamic plots of iDNA in PBMCs at each time point. The copy number was significantly reduced after Pc infection in the ART + Pc group (under ART) compared with the ART group (1.11 ± 6.76 vs. 0.23 ± 10.05 copies/10^5^ PBMCs). The meaning of each arrow is the same as indicated in Figure 1. Fisher’s exact test was utilized to examine the difference in IUPM between the two monkey groups. The numbers of SIV iDNA copies at weeks 51, 54 and 73 in the PBMCs of monkeys in the same group were combined to examine the differences between the two groups. The Mann–Whitney U test was utilized. Empower (R) (www.empowerstats.com, X&Y Solutions, Inc., Boston, MA) and R (http://www.R-project.org) were used for smooth curve fitting.

Pc infection significantly decreased the frequency of resting CD4+ T cells harboring replication-competent virus (defined as infectious units per million cells, or IUPM) (Figure 2A and B, Table 1). After the malaria was treated, the IUPM in the ART + Pc group remained significantly lower at weeks 68 and 73 compared with the IUPM in the ART group before ART was terminated (“No-malaria phase,” as shown in Figure 1; *P =* 0.001; Figure 2B). The ART + Pc group also showed a lower iDNA load in PBMCs after Pc infection (under ART) compared with the load in the ART group (means of 0.23 and 1.11 copies per 10^5^ PBMCs, respectively; *P =* 0.047; Figure 2C). These results suggested that under ART, Pc infection decreased the viral reservoirs in SIV-infected macaques.

**Table 1:** **Resting CD4+ T cells’ IUPM for each monkey**

**Monkey grouping and ID**		**Phase, week post-SIV inoculation, and IUPM**					
		**ART phase**		**Malaria phase**		**No-malaria phase**	
		**Week 43**	**Week 46**	**Week 58**	**Week 63**	**Week 68**	**Week 73**
ART	26	1.6	0.51	1.6	1.6	1.6	0.51
	28	0.51	0.51	1.6	1.6	0.51	1.6
	30	0.51	0.51	1.6	1.6	1.6	0.51
	32	3.2	3.2	1.6	1.6	0.51	1.6
	34	1.6	1.6	1.6	1.6	*	0.51
	36	1.6	1.6	0.51	0.51	3.2	1.6
ART + Pc	27	3.2	1.6	0.51	0.51	*	<0.51
	29	0.51	0.51	<0.51	<0.51	0.51	0.51
	31	1.6	1.6	<0.51	<0.51	0.51	<0.51
	33	3.2	3.2	1.6	0.51	<0.51	<0.51
	35	1.6	1.6	<0.51	<0.51	<0.51	0.51
	37	3.2	0.51	<0.51	<0.51	0.51	<0.51

*Sorted resting CD4+ T cells were insufficient for co-culture assays; therefore, no data were obtained. The detection limit was 0.51, and IUPM values less than the detection limit are indicated as “<0.51” or defined as the most likely largest value (0.32) to formulate the fitted curve in Figure 2B.

### Pc infection increased the apoptosis of memory CD4+ T cells

**Figure 3:**
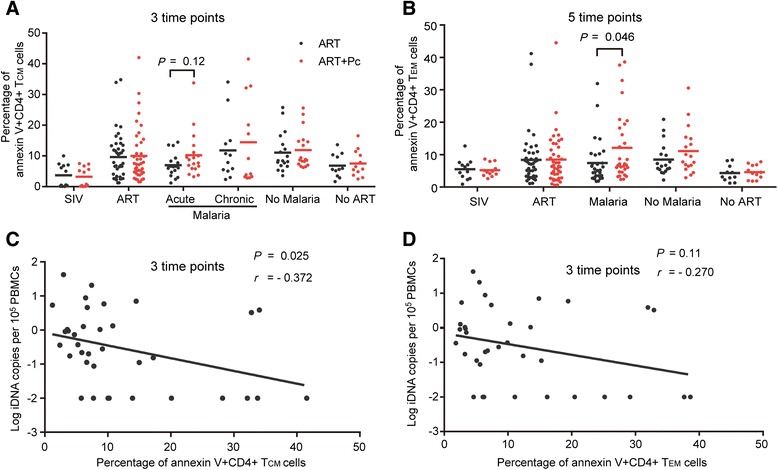
**Pc infection induced CD4+ TCM and TEM cell apoptosis during the malaria phase. (A)** During the malaria phase, the apoptosis level of CD4+ TCM cells in the ART + Pc group was higher than that in the ART group, especially during acute malaria (weeks 49–53), although this difference was not significant (10.24 ± 7.34% vs. 6.98 ± 3.77%, P = 0.12; perhaps the limited significance was a result of the small number of monkeys). **(B)** Pc infection induced higher levels of apoptosis of CD4+ TEM cells throughout the malaria phase (12.09 ± 10.58% vs. 7.46 ± 6.81%). **(C)** A negative correlation was noted between the percentage of apoptotic CD4+ TCM cells and the SIV iDNA load in PBMCs during the malaria phase. **(D)** An approximate negative correlation was noted between the percentage of apoptotic CD4+ TEM cells and the SIV iDNA load in PBMCs during the malaria phase. Variables at different time points in the same phase and group were combined for statistical analyses. The Mann–Whitney U test was utilized. Pearson’s correlation analysis was used to analyze the relationship between the parameters tested in **C** and **D**.

Induction of memory-cell apoptosis potentially leads to reduction of SIV reservoirs in rhesus macaques [16]. To determine whether Pc infection affects CD4+ T_CM_ and T_EM_ cell viability, we measured the percentages of CD4+ T_CM_ and T_EM_ cells with annexin V expressed on the cell membrane. The marker Ki67 was also used to evaluate the proliferation of these cells. CD28 + CD95 + CD4+ T cells were defined as CD4+ T_CM_ cells, CD28 + CD95-CD4+ T cells were defined as CD4+ T_EM_ cells in rhesus macaques according to previously report [17]. Our results suggested that Pc infection did not increase the proliferation levels of CD4+ T_CM_ and T_EM_ cells (data not shown). However, the apoptosis levels of CD4+ T_CM_ cells in the ART + Pc group were higher than those in the ART group during the malaria phase (especially during the acute phase, from weeks 49–53; means of 8.25% and 6.56%, respectively; *P =* 0.12; Figure 3A). There were also significantly more apoptotic CD4+ T_EM_ cells in the ART + Pc group than in the ART group (means of 12.09% and 7.46%, respectively; *P =* 0.046; Figure 3B). We also observed that the number of iDNA copies in PBMCs during the malaria phase was negatively correlated with the apoptosis levels of CD4+ T_CM_ cells in the two groups (*P* = 0.025, *r* = −0.372; Figure 3C). Additionally, the number of iDNA copies was negatively correlated with the apoptosis levels of CD4+ T_EM_ cells (*P* = 0.11, *r* = −0.270, respectively; Figure 3D). These results suggested that the reduction of the viral reservoir in the ART + Pc group might have been associated with the increased levels of CD4+ T_CM_ and T_EM_ cell apoptosis during the course of malaria.

### Pc infection selectively activated the immune system in SIV-positive monkeys under ART, which potentially led to memory CD4+ T cell apoptosis and a reduction of the SIV reservoir

**Figure 4:**
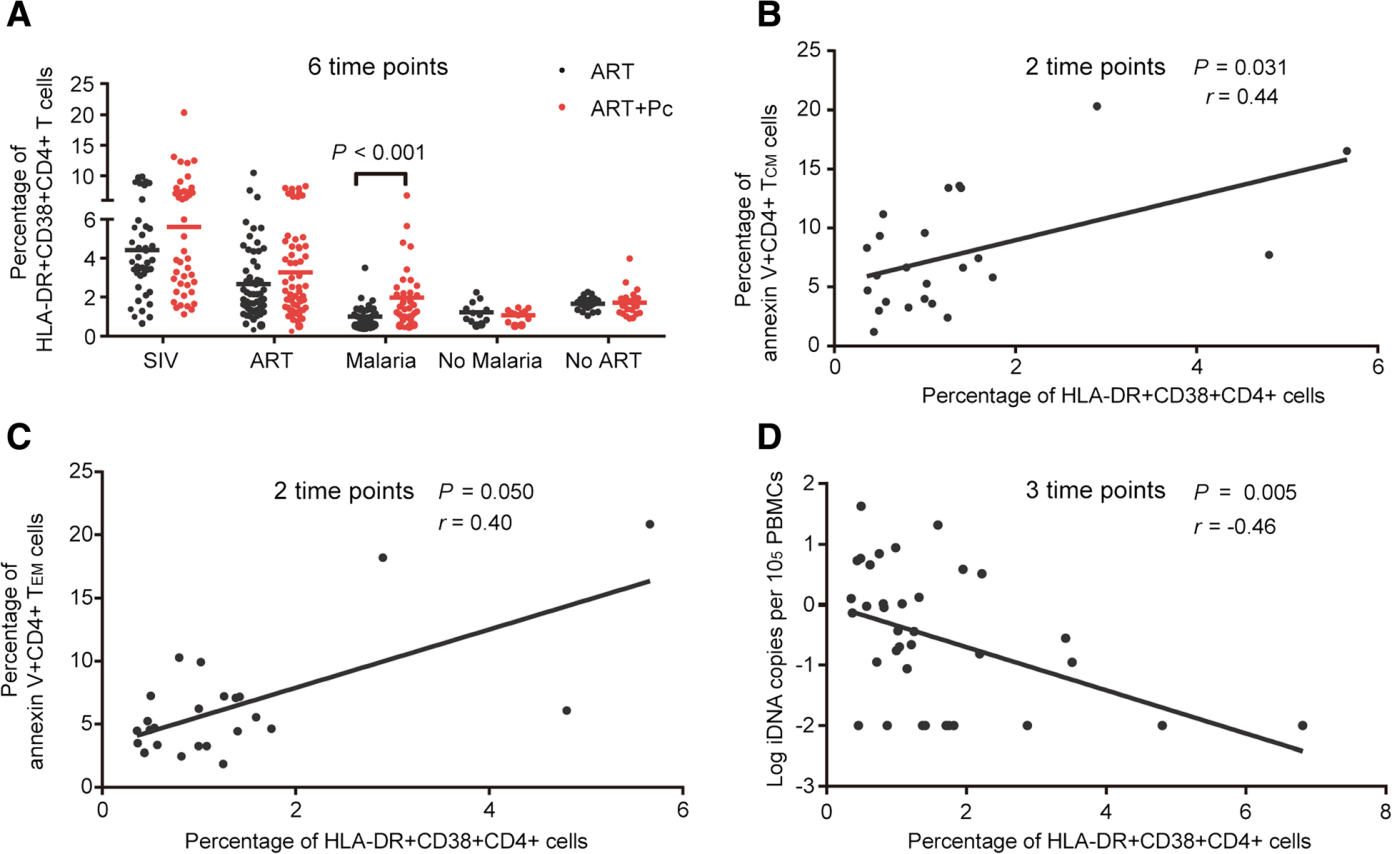
**The activation of CD4+ T cells during malaria infection potentially resulted in the apoptosis of CD4+ T**_**CM**_ **and T**_**EM**_ **cells, leading to a reduction of the SIV reservoir. (A)** Pc infection induced the co-expression of HLA-DR and CD38 in CD4+ T cells, which indicated the activation of these cells (1.97 ± 1.47% vs. 1.00 ± 0.61%). **(B)** A significant positive correlation was noted between the percentage of HLA-DR + CD38 + CD4+ T cells and the percentage of apoptotic CD4+ T_CM_ cells during the malaria phase in the two groups of monkeys. **(C)** A positive correlation was noted between the percentage of HLA-DR + CD38 + CD4+ T cells and the percentage of apoptotic CD4+ T_EM_ cells during the malaria phase in the two groups of monkeys. **(D)** A significant negative correlation was noted between the percentage of HLA-DR + CD38 + CD4+ T cells and the SIV iDNA load in PBMCs. The percentages of HLA-DR + CD38 + CD4+ T cells at different time points in the same phase and group were combined to examine the difference between the two groups; the Mann–Whitney U test was utilized, and the data are shown as the mean ± SD. Spearman’s correlation analysis was used to analyze the relationship between the parameters tested in **C** and **D**.

Our previous study using the rhesus monkey model indicated that Pc infection activated CD4+ T cells in SIV-negative animals [7]. In the present study, we examined activation markers of CD4+ T cells in SIV-positive animals under ART, including HLA-DR and CD38 co-expression on CD4+ T cells and plasma cytokine concentrations. Malaria did not increase the concentrations of plasma cytokines such as TNF-α, interleukin-7 (IL-7), interleukin-2 (IL-2, represented by its activity marker, the IL-2 receptor, or IL-2R) and IL-6, which have been shown to promote provirus reactivation [18-21] and to represent activation of the immune system (Additional file 2: Figure S2A). However, CD4+ T cell activation was found in the ART + Pc group because a greater percentage of HLA-DR + CD38 + CD4+ T cells, which is a robust measure of CD4+ T cell activation, was observed (Figure 4A). Higher concentrations of plasma neopterin (an activity marker of interferon-gamma, or IFN-γ) were also observed in the ART + Pc group during malaria infection (Additional file 2: Figure S2B). There was a significant positive correlation between plasma neopterin levels and the percentage of HLA-DR + CD38 + CD4+ T cells during the malaria phase (*P =* 0.002; *r* = 0.493; Additional file 2: Figure S2C), suggesting that the activation of CD4+ T cells was associated with higher concentrations of neopterin induced by malaria. Moreover, the CD4+ T cell activation level, as indicated by the percentage of HLA-DR + CD38 + CD4+ T cells, was shown to be positively correlated with the percentage of annexin V + CD4+ T_CM_ and T_EM_ cells during the malaria phase (*P* = 0.031, *r* = 0.44 and *P* = 0.050, *r* = 0.404; respectively; Figure 4B and C). The CD4+ T cell activation level during the malaria phase was also negatively correlated with the iDNA levels in PBMCs (*P* = 0.005, *r* = −0.458; Figure 4D). Additionally, a greater percentage of CCR5 + CD4+ T cells, which have also been linked to the activation of CD4+ T cells and the immune system [22,23], was observed in the ART + Pc group during malaria infection (Additional file 2: Figure S2D). These results suggested that malaria activated CD4+ T cells, which might have led to the apoptosis of certain CD4+ T_CM_ and T_EM_ cells and contributed to the reduction of the SIV reservoir.

### Pc infection induced histone acetylation in latently SIV-infected resting CD4+ T cells and reactivated the provirus in these cells

**Figure 5:**
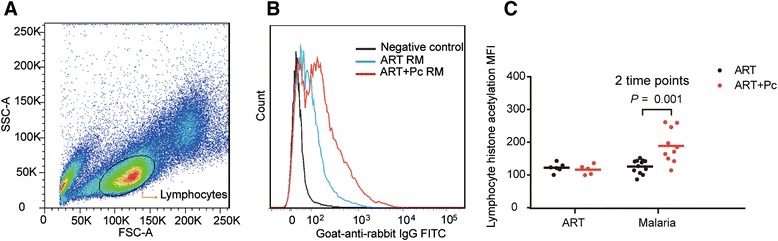
**Flow cytometric quantitation of the histone acetylation levels in lymphocytes from different phases in the two groups of monkeys. (A)** Lymphocytes gated by size on a forward scatter vs. side scatter plot. **(B)** Two monkeys were selected from different groups for representative flow cytometric analyses of different histone acetylation levels. The acetylated histone levels of the gated cells were analyzed by measuring the fluorescence intensity of these cells. The figure presents the difference in the lymphocytes’ acetylation levels in the different groups at week 54 (5 weeks after Pc infection). **(C)** Higher histone acetylation levels were observed in lymphocytes in the ART + Pc group during Pc infection (MFI of 188.90 ± 51.33 at week 54 vs. 125.96 ± 20.80 at week 57). MFI values from weeks 54 and 57 in the same group were combined to examine the difference in acetylated histone levels between the two groups during the malaria phase. The Mann–Whitney U test was utilized for statistical analyses. The data are shown as the mean ± SD.

Previous studies showed that histone acetylation in resting CD4+ T cells could induce reservoir purging [24,25]. We thus examined the histone acetylation levels in peripheral blood lymphocytes (PBLs) in the monkeys during different phases. The results showed that the levels of global histone acetylation in PBLs, as measured based on mean fluorescence intensity (MFI), were much higher in the ART + Pc group during Pc infection than the levels in the ART group (MFI of 188.90 vs. 125.96; *P =* 0.001; Figure 5A–C).

**Figure 6:**
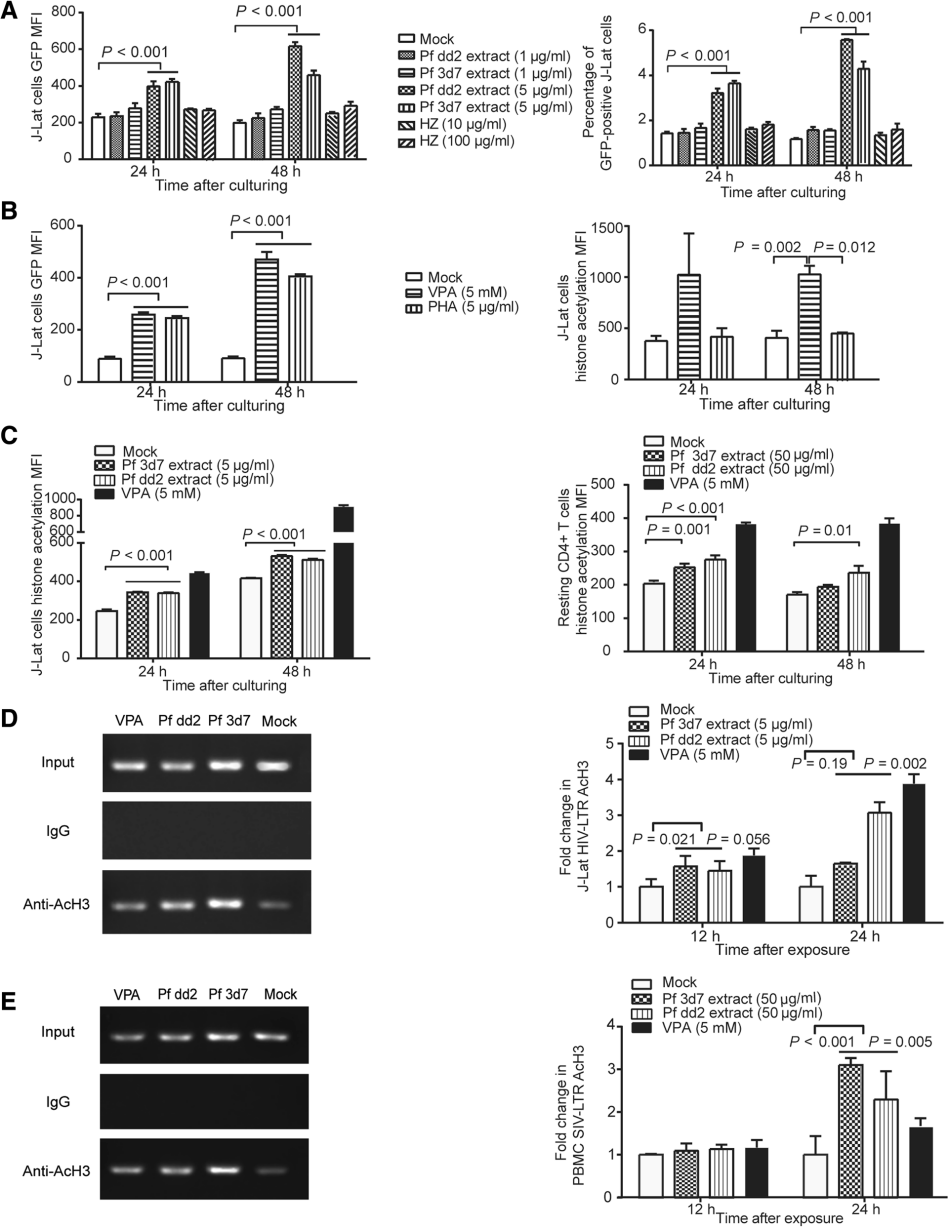
***In vitro*** **assays measuring activation and histone acetylation of the HIV-1 or SIV promoter by Pf extracts. (A-B)** The effects of the synergistic activation of the HIV-1 promoter were determined by quantifying GFP-positive cells using flow cytometry 24 or 48 hours after treatment. **(A)** In total, 5 μg/ml Pf soluble extract induced HIV-1 promoter activation in J-Lat cells, leading to GFP expression. **(B)** Both PHA and VPA induced activation of the HIV-1 promoter in J-Lat cells and subsequent GFP expression. In contrast, PHA did not induce increased levels of histone acetylation in J-Lat cells, whereas VPA induced histone acetylation. **(C)** 5 μg/ml *Plasmodium* soluble extract increased histone acetylation levels in J-Lat cells, and 50 μg/ml *Plasmodium* soluble extract was sufficient to induce increased levels of histone acetylation in resting CD4+ T cells. This result was obtained from FACS assays. **(D-E)** Histone acetylation modifications in the HIV-1 LTR and SIV LTR promoters induced by *Plasmodium* soluble extract. Chromatin fragments from J-Lat cells or monkey PBMCs cultured for 24 hours with or without *Plasmodium* soluble extract or VPA were immunoprecipitated with anti-AcH3 or with normal rabbit serum (IgG) as a control. PCR was then used to amplify the DNA isolated from the immunoprecipitated chromatin. The relative dilution ratio of input/chip was 1.33. **(D)** Pf soluble extract induced an increase in the histone acetylation levels in the HIV-1 LTR. **(E)** Pf soluble extract induced an increase in the histone acetylation levels in the SIV LTR. The left panels of D and E show semi-quantitative results based on PCR products using input DNA, normal IgG control product or 24-hour CHIP product as the template. The data in this figure are presented as the means, and the error bars indicate the SD. One-way ANOVA was used to compare the variables in this figure.

We further explored whether the products of malaria parasites participated in the purging of the viral reservoir. A chloroquine-sensitive strain (3d7) and a chloroquine-resistant strain (dd2) of the human malaria parasite *Plasmodium falciparum* (Pf) were used for these tests. In particular, we prepared the Pf 3d7 metabolite hemozoin (HZ) and soluble extracts to test their potencies in reactivating latent cells. An *in vitro* assay was then performed with J-Lat 10.6 cells to determine whether Pf HZ or soluble extract could reactivate these latently infected CD4+ T cells by observing the expression of HIV-1-pseudotyped virus. The results showed that 5 μg/ml Pf soluble extract could reactivate the J-Lat 10.6 cells. Increased GFP expression after 24–48 hours of co-incubation was observed, indicating the expression of latent virus. However, 10–100 μg/ml HZ did not activate the J-Lat 10.6 cells (Figure 6A). Additionally, valproic acid (VPA) and phytohemagglutinin (PHA) were used as positive controls. Both compounds induced the expression of latent virus in J-Lat 10.6 cells, but only VPA induced more acetylated histones (Figure 6B). Fluorescence-activated cell sorting (FACS) assays also showed that 5 μg/ml Pf soluble extract could induce more histone acetylation in J-Lat 10.6 cells after 24- or 48-hour co-incubation. In resting CD4+ T cells isolated from the PBMCs of the SIV-infected monkeys, 50 μg/ml Pf soluble extract could also induce histone acetylation after a 24- or 48-hour co-incubation (Figure 6C). In contrast, 1 μg/ml Pf soluble extract did not induce much more histone acetylation in J-Lat 10.6 cells (Additional file 3: Figure S3), and 25 μg/ml Pf soluble extract dramatically induced the death of J-Lat 10.6 cells (data not shown). Unlike the situation in J-Lat cells, 50 μg/ml Pf soluble extract could sufficiently induce significantly more histone acetylation in the monkey resting CD4+ T cells (Additional file 3: Figure S3). These results indicated that the sensitivity of the J-Lat cell line and primary monkey cells to Pf extract was not the same.

Chromatin immunoprecipitation (ChIP) assays showed that the amounts of acetylated histone H3 (AcH3) bound to the core promoter region within the HIV-1 and SIV long terminal repeats (LTRs) increased when cells were co-incubated with Pf soluble extract Figure 6D and E). In particular, in J-Lat 10.6 cells, the acetylated histone levels increased 1.65-fold when the cells were stimulated with Pf 3d7 extract and increased 3.07-fold when the cells were stimulated with Pf dd2 extract. In contrast, in monkey PBMCs, the levels increased 3.10-fold with Pf 3d7 extract and 2.30-fold with Pf dd2 extract after a 24-hour co-incubation (Figure 6D and E).

### Pc infection might activate the NF-κB signaling pathway in latently infected CD4+ T cells

**Figure 7:**
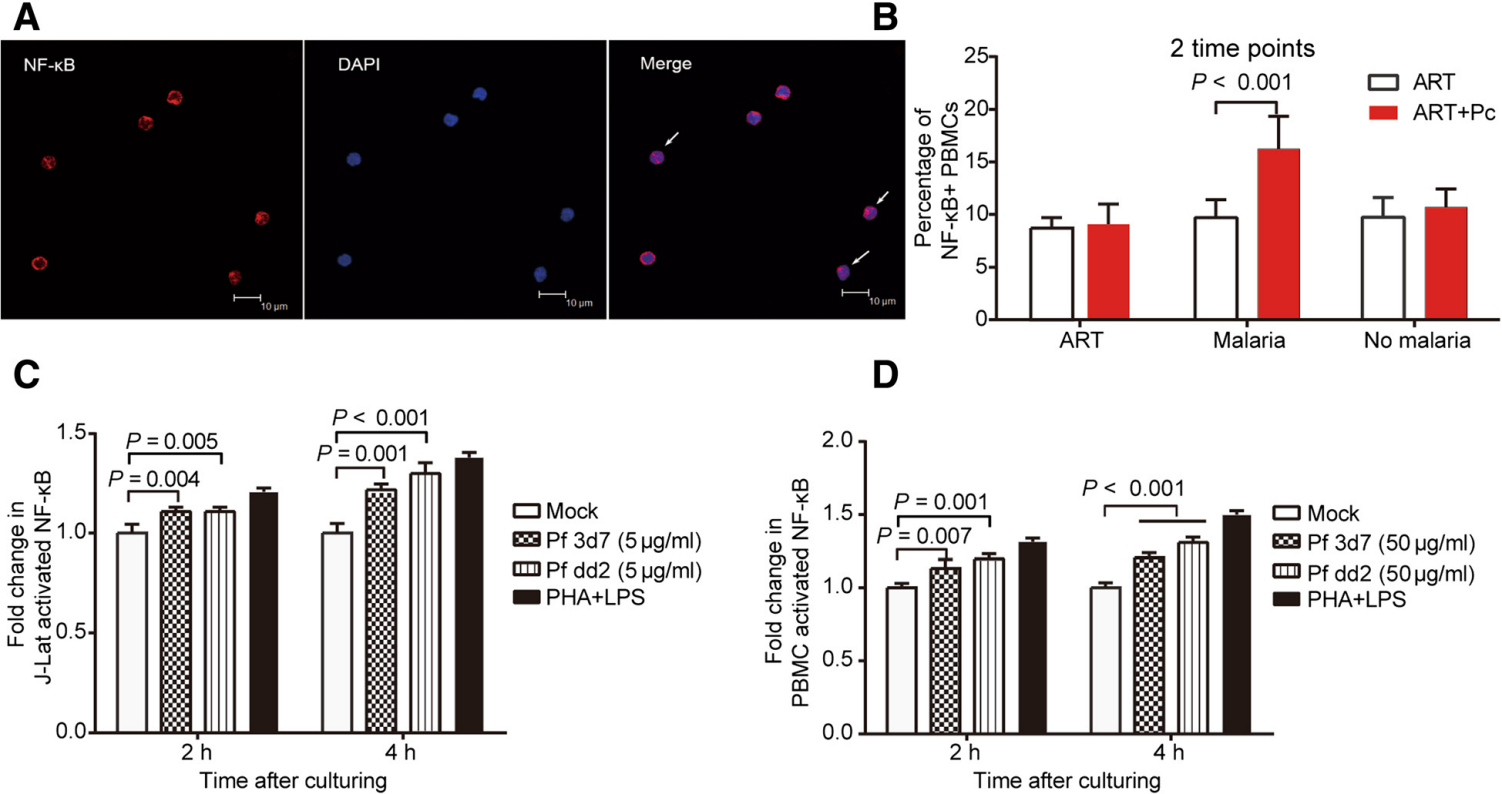
***In vivo*** **and** ***in vitro*** **assays for NF-κB activation. (A–B)** An *in vivo* assay as performed to measure NF-κB activation levels during malaria infection. In particular, PBMCs were isolated from monkeys in the two groups at different time points for each phase. We counted the cells with NF-κB nuclear staining and classified them as cells with NF-κB activation. **(A)** NF-κB nuclear transport was measured by immunofluorescence to determine the activation of NF-κB in PBMCs. DAPI staining was used to identify the nuclear region to assess gross cell morphology. The white arrows show the NF-κB-positive nuclei in PBMCs. These cells were considered to contain activated NF-κB. **(B)** The ART + Pc group maintained higher levels of NF-κB activation in PBMCs during malaria infection, as indicated by the percentages of NF-κB-positive nuclei in the PBMCs (16.23 ± 3.12% vs. 9.69 ± 1.74%). The data in this figure are presented as the means, and the error bars show the SD. **(C-D)** *In vitro* assays were performed to detect specific factors associated with malaria that activate NF-κB. PBMCs isolated from monkeys were stimulated with Pf extract or 25 ng/ml PHA plus 1 μg/ml LPS. NF-κB activation in the cells was then measured by ELISA. **(C)** Pf soluble extract induced an increase in activated NF-κB in J-Lat cells at 5 μg/ml after a 2- or 4-hour incubation. **(D)** Pf soluble extract induced an increase in activated NF-κB in RM PBMCs at 50 μg/ml after a 2- or 4-hour incubation. The percentages of NF-κB+ PBMCs at different time points of the same phase in the same group were combined for statistical analyses. The Mann–Whitney U test was utilized to examine the difference in this parameter between the two groups. One-way ANOVA was used to compare the variables in C and D. The data in this figure are presented as the means, and the error bars indicate the SD.

Given that previous studies demonstrated that NF-κB activation was associated with provirus reactivation [26,27], we investigated NF-κB translocation in monkey PBMCs in selected phases. This experiment used immunofluorescence confocal microscopy to assess the activation level of NF-κB. The percentage of NF-κB+ cells was significantly higher in the PBMCs of monkeys in the ART + Pc group during *Plasmodium* infection compared with the percentage in the ART group (means of 16.23% and 9.69%, respectively; *P <* 0.001; Figure 7A and B). This result suggested that the NF-κB signaling pathway might participate in the latent virus reactivation induced by malaria. An *in vitro* culture assay also indicated that 5 μg/ml or 50 μg/ml *Plasmodium* extract could induce NF-κB activation in J-Lat cells and monkey PBMCs (Figure 7C and D).

### Memory CD4+ T cell activation, apoptosis and latent virus reactivation induced by Pc infection were partly associated with *Plasmodium* extract stimulation

Our *in vivo* study showed that *Plasmodium* infection caused CD4+ T cell activation and might have led to the apoptosis of memory CD4+ T cells and a decrease in the iDNA load in PBMCs. The *in vitro* study also showed that *Plasmodium* extract could activate latently infected J-Lat 10.6 cells and cause the expression of latent virus (Figure 6A). To test the potency of *Plasmodium* extract in activating latently SIV-infected memory CD4+ T cells and inducing the apoptosis of these cells, we performed an *in vitro* assay in which PBMCs from a chronically SIV-infected monkey were treated with different concentrations of Pf 3d7 or dd2 extract.

**Figure 8:**
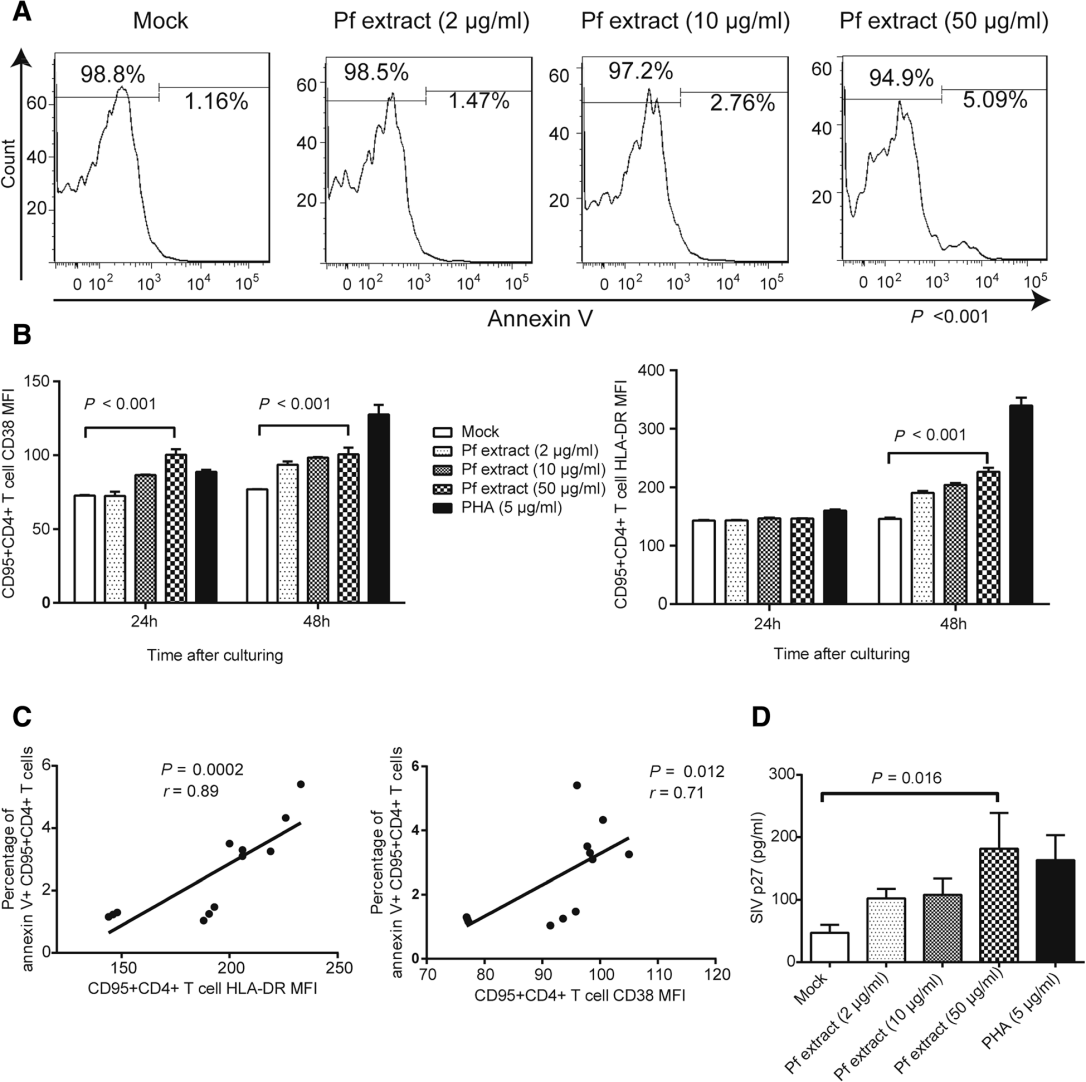
***In vitro*** **assays measuring the apoptosis and activation of monkey memory CD4+ T cells by Pf extract.** PBMCs from a monkey with chronic SIV infection were isolated and cocultured with Pf extract. After coculture for 24 or 48 hours, the cells were stained with anti-CD4 PE-Cy7, anti-CD95-APC, and annexin V PE or with anti-CD4 PE-Cy7, anti-CD95 APC, anti-CD38 FITC and anti-HLA-DR PE. Memory CD4+ T cells were gated (CD95 + CD4+ T lymphocytes) and examined for the percentages of cells that stained with annexin V. Alternatively, the MFI of CD38 and HLA-DR was measured. **(A)** Both 10 μg/ml and 50 μg/ml Pf extract could dramatically induce the apoptosis of memory CD4+ T cells after 48 hours of coculture (3.30 ± 0.20% vs. 1.23 ± 0.07% and 4.36 ± 1.10% vs. 1.23 ± 0.07%, respectively, *P* < 0.001). **(B)** In this study, 50 μg/ml Pf extract could induce the activation of memory CD4+ T cells, leading to more CD38 (MFI of 100.50 ± 4.50 vs. 75.27 ± 1.25) and HLA-DR (MFI of 226.00 ± 7.00 vs. 146.00 ± 2.000) expression on these cells after 48 hours of coculture. **(C)** A positive correlation was found between the memory CD4+ T cells’ activation level and apoptosis level. **(D)** In this study, 50 μg/ml Pf extract could induce more SIV p27 antigen expression after 8 days of coculture (181.70 ± 57.08 vs. 47.11 ± 12.75 pg/ml). The data in this figure are presented as the means with SD. One-way ANOVA was used to compare the variables in A, B and D. Spearman’s correlation analysis was used to analyze the relationship between the parameters in C.

Figure 8A and B show the potency of Pf 3d7 extract in inducing the activation and apoptosis of monkey memory CD4+ T cells. The results showed that Pf extract could induce the apoptosis of memory CD4+ T cells and the activation of these cells. Specifically, more expression of the activation marker CD38 and HLA-DR was found after co-incubation with Pf extract (Figure 8A and B). Correlation analysis showed that the apoptosis of memory CD4+ T cells positively correlated with the activation level of these cells (Figure 8C). Pf dd2 extract had similar potency in inducing the activation and apoptosis of monkey memory CD4+ T cells (data not shown). In addition, more expression of the SIV antigen p27 protein was induced after 8 days of culture with Pf extract (Figure 8D).

## Discussion

Because malaria infection can induce CD4+ T cell activation and lead to the replication of HIV-1 in patients without ART [9], we postulated that malaria might activate resting CD4+ T cells, thereby promoting HIV-1 transcription and replication. This viral reactivation would then purge the HIV-1 reservoir in patients undergoing ART. We utilized a monkey model to test this hypothesis.

Rhesus macaques provide an ideal model for HIV-1 and malaria infection in humans [28-30]. SIV-infected rhesus macaques have revealed key aspects of HIV-1 pathogenesis, such as virus transmission, early post-infection events, the sites of viral replication, CD4+ T cell depletion, and virus and cell turnover [31], and these animals have been utilized in studies of viral reservoirs [16]. Compared with Chinese rhesus macaques, the Indian rhesus macaque model has limitations [32,33]; SIV pathogenesis in Chinese rhesus macaques more closely resembles HIV-1 infections in humans [34]. Beyond *P. cynomolgi* (Pc), two other malaria parasites, *Plasmodium knowlesi* and *Plasmodium coatneyi*, which can also infect rhesus macaques, are fulminating in rhesus macaques and nearly always result in death [35]. Thus, we utilized Pc and SIVmac251 co-infection in Chinese rhesus macaques to mimic HIV-1 and malaria co-infection in humans.

Malaria infection can increase the plasma viral load in HIV-1-infected individuals and in monkey models without ART [9,14]. In the present study, the ART + Pc group showed a 0.27-log higher plasma viral load during malaria infection compared with the load in the ART group (2.01 log vs. 1.74 log; *P* = 0.090; Additional file 1: Figure S1D). Because the iDNA level in PBMCs was lower in the ART + Pc group during malaria infection, this small change in plasma virus levels might not have been derived from viral replication in pre-existing activated CD4+ T cells or in newly infected CD4+ T cells, which are both susceptible to SIV because of the higher level of CCR5 expression on CD4+ T cells (Additional file 2: Figure S2D). This result also suggested that the ART regimen in this study effectively blocked SIV entry into these susceptible cells. A reasonable explanation is that this small increase in virus levels might have been derived from the activation of latently infected cells, which was reflected by our *in vivo* finding that malaria increased the activation level of CD4+ T cells (Figure 4A) and our *in vitro* finding that *Plasmodium* extract could induce activation of the main cellular reservoir, the memory CD4+ T cells (Figure 8B). The higher levels of IFN-γ activity (represented by higher levels of plasma neopterin) induced by malaria also indicated immune system and T cell activation [13]. The higher levels of neopterin induced by malaria especially indicated the activation of macrophages [36], which may be more critical in the process of SIV reservoir purging because Van der Sluis *et al*. have shown that APCs such as myeloid dendritic cells can activate latent T cells more than other T cell activators do [37]. A correlation analysis of plasma neopterin and the activation level of CD4+ T cells might also have uncovered this phenomenon (Additional file 2: Figure S2D). In contrast to neopterin, neither the expression of the other cytokines tested, including IL-2, IL-6, IL-7 and TNF-α, nor their activities increased during malaria infection under ART (i.e., there were no differences between the two groups; Additional file 2: Figure S2A), indicating that ART potentially alters the interaction of SIV and the malaria parasite *in vivo*.

The activation of resting CD4+ T cells could facilitate the apoptosis of these cells [38,39]. Malaria infection specifically induces apoptosis in mononuclear cells, including memory T cells, and contributes to reduction of the immune response to critical antigens [40]. This effect was also observed in the ART + Pc group (Figure 3A and B) and in our *in vitro* assay (Figure 8A). However, due to the relatively small number of animals in the present study, we did not observe a significant effect of malaria on CD4+ T_CM_ cell apoptosis, but monkeys that were co-infected with malaria did maintain a relatively higher level of apoptotic CD4+ T_CM_ cells (Figure 3A). Moreover, the effect of malaria on CD4+ T_EM_ cell apoptosis was significant (Figure 3B). We also observed significantly high levels of apoptotic memory cells induced by Pf extract in our *in vitro* study (Figure 8A). These results indicated that malaria did induce the apoptosis of memory CD4+ T cells, which were the main cellular reservoir of SIV. It is possible that several “long-lived” T_CM_ cells can transition into “short-lived” T_EM_ cells that are more susceptible to apoptotic signals [41]. Thus, a significantly higher level of apoptotic T_EM_ cells was observed, but the final outcome might have been the result of the “indirect apoptosis” of T_CM_ cells. All of these effects could be associated with the expression of the activation markers HLA-DR and CD38 on CD4+ T cells, which reflects the activation level of these cells (Figure 4A–C). Furthermore, the reduction of the SIV iDNA load in PBMCs was significantly negatively correlated with increased levels of apoptotic CD4+ T_CM_ cells and the proportion of HLA-DR + CD38 + CD4+ T cells during malaria infection (Figures 3C and 4D). We also found that the SIV iDNA load in PBMCs negatively correlated with increased levels of apoptotic CD4+ T_EM_ cells although no significant and this may due to the small number of animals in present study (Figure 3D). These results indicated that malaria infection activated and induced the apoptosis of CD4+ T cells, including memory cells, thereby leading to the reduction of the SIV reservoirs. The *in vitro* study also demonstrated that Pf extract could activate the memory CD4+ T cells, along with the production of virion, and could induce the apoptosis of these cells, suggesting that malaria-induced reservoir purging was partly related to the *Plasmodium* itself. This result was partly consistent with a report by Froebel *et al.*, showing that malaria antigens could reactivate the replication of endogenous HIV in cells from HIV-infected adults [13].

Histone acetylation in cells is an epigenetic change that can induce transcriptional activation of HIV-1 provirus and lead to reservoir purging [25,42]. In the present study, higher levels of histone acetylation in the PBLs of Pc-infected monkeys were observed. Activation of NF-κB is also associated with provirus reactivation [27]; we observed more NF-κB translocation in PBMCs from the Pc-infected monkeys. This result was consistent with Chuchard’s report, which showed that PBMCs in malaria patients maintained higher levels of NF-κB activation [43]. Our *in vitro* studies also confirmed that Pf extracts could induce histone acetylation in resting CD4+ T cells and in latently HIV-1-infected cells (J-Lat cells) and could activate NF-κB signaling in monkey PBMCs and in J-Lat cells. Histone acetylation in cells is not necessarily associated with the global activation of host cells, which is similar to the situation induced by the histone deacetylase (HDAC) inhibitor VPA [44]. However, reactivation of latent virus would lead to antigen expression on host cells, which would induce antigen-specific cytotoxic lymphocyte responses that would kill the infected cells [4]. Therefore, histone acetylation and NF-κB activation may also play important roles in the reduction of the SIV reservoir.

In the present study, we observed that malaria caused epigenetic changes in monkeys and that this phenomenon was related to the *Plasmodium* itself. This result was unexpected. In particular, many environmental factors, such as exercise, diet and medications, can cause epigenetic and developmental epigenetic changes [45,46], whereas our results indicated that a type of microorganism infection can cause such changes and this was related to its extract. Further experiments demonstrated that J-Lat cells and primary monkey cells had different response patterns in the presence of Pf extract and that 5 μg/ml Pf extract could induce a considerable increase in histone acetylation in J-Lat cells, whereas more Pf extract was needed to induce an increase in histone acetylation in monkey resting CD4+ T cells (Additional file 3: Figure S3). The differences in patterns might be attributable to the different proliferation levels of these two types of cells; J-Lat cells, which are derived from Jurkat cells, are actively proliferating tumor cells, and primary monkey cells are non-actively proliferating cells. The different cell cycle patterns in these two types of cells may cause different sensitivities to Pf extract, as it has been shown that different cell cycle patterns may lead to different sensitivities to certain factors [47,48]. The present study also showed that the increase in the histone acetylation level induced by Pf extract was not due to regulation of HDAC, which has been shown to participate in regulating the histone acetylation level. This finding indicated that the mechanism of *Plasmodium* inducing histone acetylation was not similar to that of an HDAC inhibitor (Additional file 4: Figure S4). However, the exact mechanism needs to be studied further and in depth. We do not know whether this phenomenon was *Plasmodium* specific, but lipopolysaccharide (LPS) did not induce an increased level of histone acetylation in primary monkey cells (Additional file 5: Figure S5), indicating that certain factors related to bacteria might not be associated with this phenomenon.

In the present study, we did not observe a delay in viral rebound in the ART + Pc group compared with the ART group when ART was terminated at week 75 after SIV inoculation (Additional file 1: Figure S1). The re-emergence or rebound of plasma viremia has also previously been observed following the cessation of ART, despite profoundly low HIV-1 reservoir levels [49]. Possible reasons for this observation in rhesus macaques include the following. First, although the cellular reservoir of SIV decreased during malaria infection, the frequency of resting CD4+ T cells harboring replication-competent SIV remained a large burden for the monkeys. Second, the possibility of the existence of low levels of SIV replication, the persistence of the virus in lymphoid tissues or cryptic viral replication in anatomical reservoirs in macaques treated with ART cannot be eliminated due to the efficiency of the ART regimen.

## Conclusions

Altogether, our results indicate that malaria reduced the volume of the SIV reservoir in monkeys that underwent ART. We have highlighted the apoptosis of memory CD4+ T cells during Pc infection, which is a potential outcome of the activation of these cells induced by malaria. Our *in vivo* and *in vitro* results indicate that histone acetylation and NF-κB activation in resting CD4+ T cells may also play important roles.

As more HIV-1-infected individuals in malaria-endemic areas receive ART, physicians and researchers should pay attention to the impact of malaria on the viral reservoir of these individuals. For example, repeated or longer courses of malaria infection may occur and more profoundly reduce the volume of the viral reservoir in HIV-1-infected individuals who are receiving effective ART in areas that are endemic for both HIV-1 and malaria. Several of these patients may experience virologic benefits. In addition, based on our previous study [50], modified malaria parasites should be explored as vectors for therapeutic HIV-1 vaccine development.

## Methods

### Animals and study design

The animals were housed and fed according to regulations established by the Guide for the Care and Use of Laboratory Animals and the Animal Welfare Act at the Non-human Primate Animal Center of the Guangzhou Institutes of Biomedicine and Health (GIBH). The animal experiments performed in this study were approved by the GIBH Institutional Animal Care and Use Committee (IACUC) (approval ID 2010008). Figure 1 shows the study design. All monkeys were treated with ART 15 weeks after SIV inoculation. The treatment regimen included 30 mg/kg 9-R-(2-phosphonomethoxypropyl) adenine (PMPA) and 20 mg/kg emtricitabine (FTC), administered once daily intramuscularly (i.m.). Then, 100 mg raltegravir (an integrase inhibitor) was added orally twice per day at week 36. At week 49, the monkeys in the ART + Pc group were intravenously inoculated with 2.38 × 10^7^ Pc-infected red blood cells (RBCs). As controls, monkeys in the ART group were inoculated with 2.38 × 10^7^ normal RBCs. The Pc infection was terminated at week 66 by oral chloroquine administration, and ART was terminated at week 75.

### Plasma SIV levels

The levels of plasma SIV RNA were measured by quantitative reverse transcriptase PCR (qRT-PCR), as previously described [51]. The number of SIV RNA copies was calculated from a regression curve derived from RNA transcript standards. This value was divided by the volume of the extracted plasma specimen to obtain a value in units of SIV RNA copy equivalents/ml of plasma. In brief, 2000 μl plasma was centrifuged at 21,000 × g for 3 hours at 4°C, and 1850 μl supernatant was removed. The virus pellet was then resuspended completely by vigorous vortexing and stored at −80°C for RNA isolation. RNA was isolated using the NucleoSpin viral RNA isolation kit (MACHEREY-NAGEL, Duren, Germany) according to the manufacturer’s instructions, except that the virus was incubated in lysis buffer for 10 minutes at room temperature. The RNA was eluted in 50 μl RNase-free H_2_O and frozen in aliquots at −80°C for subsequent qRT-PCR.

Modified real-time RT-PCR was performed to measure the conserved region of gag in the viral RNA, as described by Hofmann-Lehmann [52]. The primers and probe specific for the conserved gag region of SIVmac251 were as follows: forward, Alu1217, GCAGAGGAGGAAATTACCCAG; reverse, SIVgag, ACAATTTTACCCAGGCATTTAATGTT; and probe Alu1217-P6-carboxyfluorescein, (FAM)-TCGGGCTTAATGGCAGGTGGACA-BHQ1. The reactions were performed in 25 μl volumes containing 12.5 μl RNA-direct Realtime PCR Master Mix (Toyobo, Shanghai, China), 3 mM Mn(OAc)_2_, 300 nM forward and reverse primers, 100 nM probe and 5 μl template. The thermal cycling conditions consisted of 30 seconds at 90°C, 20 minutes at 61°C, and 30 seconds at 95°C, followed by 45 cycles of 1 seconds at 95°C, 45 seconds at 60°C and a plate read. The kinetic PCR amplification data were analyzed using Bio-Rad CFX Manager software. A standard curve for the viral RNA was then prepared using serial dilutions of a synthetic *gag* transcript in H_2_O containing 30 μg/ml transfer RNA from *Escherichia coli* (Sigma). This 544 bp RNA was transcribed *in vitro* using SP6 RNA Polymerase (Takara, Dalian, China) and an EcoRI-digested PMD-20 T plasmid into which a 461 bp fragment of SIV *gag* was inserted. The numbers of RNA copies were calculated from the concentration determined by the absorbance at 260 nm. Three replicate reactions were performed for the samples and standard. The numbers of copies of SIV RNA in the samples were calculated by interpolation of the experimentally determined quantification cycle (Cq) value for the test sample using the transcript-derived linear regression as a standard curve.

### Real-time qPCR for SIV iDNA in PBMCs

The amount of SIV iDNA was quantified using modified Alu-PCR according to the method of Norio *et al*. [53]. The primers and probe for the first-round PCR were mAlu-196 F20, CAGGAGAATCGCTTGAACCC; mAlu-228R19, GATCTCGGCTCACTGCAAC; and Alu1217-RArt, CAATATCATACGCCGAGAAATGTTCTCGGGCTTAATGG. The reactions were performed in 70 μl volumes containing 25 μl Premix Ex Taq (Takara), 450 nM each primer and 7 μl template. We pre-amplified a 50 μl reaction mixture under the following thermal cycling conditions: 60 seconds at 95°C, followed by 12 cycles of 10 seconds at 98°C, 30 seconds at 60°C and 5 minutes at 72°C. The rest of the reaction mixture was stored at 4°C as “non-pre-amplified” samples. The second-round real-time quantitative multiplex PCR was performed using 2 μl of the material from the pre-amplified or matched mixture of “non-pre-amplified” samples. The sequences of the primers and probes were as follows: GAPDH-cF, CCCCATAGGCGAGATCCC; GAPDH-cR, CCTCCTGCACTCACCCC; GAPDH-cP, HEX-CCACGACGTACTCAGCGCCAGCAT-BQ1; Alu1217-F, GCAGAGGAGGAAATTACCCAG; Alu1217-Art, CAATATCATACGCCGAGAAATGT; and Alu1217-P, FAM-TCGGGCTTAATGGCAGGTGGACA-BQ1. The reactions were performed in 20 μl volumes containing 10 μl THUNDERBIRD Probe qPCR Mix (Toyobo), 300 nM GAPDH-cF and GAPDH-cR, 100 nM GAPDH-cP, 450 nM Alu1217-F and Alu1217-Art, 150 nM Alu1217-P and 2 μl template. The thermal cycling conditions consisted of 60 seconds at 95°C, followed by 45 cycles of 15 seconds at 95°C, 15 seconds at 60°C and a plate read. The kinetic PCR amplification data were analyzed using Bio-Rad CFX Manager software. A standard curve for the viral DNA was prepared using serial dilutions of cellular DNA extracted from CEMss/SIV/rc cells diluted in 10^4^ cells/μl CEMss DNA. Three replicate reactions were performed for both species and the standard. The numbers of copies of SIV and cell genomes in the test samples were calculated by interpolation of the experimentally determined Cq value for the test sample using the linear regression as a standard curve. The calculated number of copy equivalents per reaction mixture is expressed as the number of SIV copies per 10^5^ cells.

### Measurement of the frequency of resting CD4+ T cells harboring replication-competent virus

Macaque resting CD4+ T cells were isolated as described elsewhere [30,54]. Briefly, macaque blood was centrifuged through discontinuous density gradients to obtain PBMCs using 95% Lymphoprep (Fresenius Kabi Norge AS, Halden, Norway). The PBMCs were labeled with FITC-conjugated anti-CD4 and APC-conjugated anti-HLA-DR (BD Biosciences, Franklin Lakes, USA) and sorted for small HLA-DR-CD4+ cells using a FACSAria II. Populations of small HLA-DR-CD4+ cells were isolated with 98–99% purity. The frequency of resting CD4+ T cells harboring replication-competent virus was then measured using a co-culture assay in which resting CD4+ T cells were cultured with the cell line CEM × 174. This co-culture both activated the macaques’ resting CD4+ T cells through CD2-CD58 interactions and expanded virus released from latently infected cells that became activated, allowing quantification of the size of the reservoir, as previously described [55,56]. The resting CD4+ T cells were cultured in a fivefold dilution series in duplicate, ranging from 1 × 10^6^ to 3.2 × 10^2^ cells per well. The presence of replication-competent SIV was then determined by observing SIV outgrowth with p27 after 3 weeks of culture. The co-culture assays were set up in a limiting-dilution format, and the frequencies of resting CD4+ T cells harboring replication-competent virus were determined using the maximum likelihood method, as previously described [56]. The lower limit of detection was 0.51 IUPM in the present study.

### T cell assays, flow cytometry, and monoclonal antibodies (mAbs)

The following mAbs were used for flow cytometry: anti-CD3 PerCP, anti-CD4 FITC, anti-CD4 PerCP, anti-CD4 PE-Cy7, anti-CD8 PerCP, anti-CD8 APC-Cy7, anti-CD28 FITC, anti-CD95 APC, anti-CD195 (anti-CCR5) PE, anti-Ki67 PE, anti-HLA-DR APC, and anti-HLA-DR FITC (BD Biosciences). Additionally, annexin V PE was purchased from BD Biosciences, anti-CD8 APC and anti-CD8 PE were obtained from Beckman Coulter (CA, USA), and anti-CD38 FITC was purchased from StemCell Technologies (Vancouver, Canada). Flow cytometry data were acquired on a FACSAria II (BD Biosciences). All data were analyzed using FlowJo software (Tree Star, Ashland, OR, USA).

### ELISA for plasma cytokines

Plasma IL-2R, neopterin, IL-6, TNF-α and IL-7 concentrations were assayed by ELISA kits according to the manufacturers’ instructions. The kits were obtained from BioLegend (for IL-2R), Groundwork Biotechnology Diagnosticate and Alpha Diagnostic International (for neopterin), Mabtech (for IL-6 and TNF-α) and R&D Systems (for IL-7). Each plasma sample was analyzed in triplicate.

### *In vivo* NF-κB activation and nuclear transport tests

NF-κB activation and nuclear transport tests were performed using a Nuclear Translocation Assay Kit (catalog no. SN368, Beyotime, Beijing, China) according to the manufacturers’ instructions. Briefly, PBMCs from monkeys were collected and then washed once with PBS. These cells were fixed, transferred to slides, and washed twice with PBS with Tween-20 (PBST). The cells on the slides were blocked using PBS with 8% BSA for 1 hour. Next, the cells were incubated with a rabbit anti-human NF-κB p65 antibody (Beyotime, Beijing, China) at 4°C overnight. The cells were then stained with a Cy3-labeled anti-rabbit antibody (Beyotime, Beijing, China), washed with PBST three times and stained with DAPI at room temperature for 10 minutes. Finally, the cells were washed with PBST three times and observed using a confocal microscope (ZEISS LSM 710). A total of 500 cells on each slide were counted. We specifically counted cells with NF-κB nuclear staining and classified them as cells with NF-κB activation.

### Measurement of global histone acetylation by flow cytometry

This assay was performed according to the method reported by Archin *et al.* [25]. Briefly, cells were fixed and permeabilized with Phosflow Fix Buffer I and Phosflow Perm Buffer II (BD Biosciences) according to the manufacturer’s protocol. The cells were then washed in staining buffer (PBS with 2% FBS and 0.1% sodium azide), blocked with 8% normal goat serum (Invitrogen), and incubated with anti-AcH3 (1:200 dilution; catalog no. 06–599, Millipore) or control rabbit IgG in blocking solution for 60 minutes at room temperature. Next, the cells were washed twice with staining buffer and incubated with goat-anti-rabbit IgG FITC- or Cy3-conjugated secondary antibody (1:200 dilution; Millipore) in staining buffer for 30 minutes at room temperature in the dark. Following a final wash, the cells were analyzed by flow cytometry using a FACSAria II flow cytometer and FlowJo software (Tree Star).

### *In vitro* experimental design, cells, culture conditions, and cell treatments

Pf 3d7 or dd2 extract or HZ was added to J-Lat cell cultures to monitor whether the treatment reactivated provirus expression, as indicated by increased GFP expression and induced histone acetylation or NF-κB activation. We also performed the same culture assays to monitor the effect of Pf extracts on resting CD4+ T cells or PBMCs from rhesus macaques. Pf extract preparation was performed using saponin to release parasites and an ultrasonic cell disruptor (Bioruptor UCD-300, Diagenode, Liège, Belgium) to extract. The concentration was measured based on the protein concentration. *Plasmodium* HZ extraction was performed as follows. Parasites were released from RBCs by 0.01% saponin lysis and were washed 5–8 times to remove RBC debris. Next, the parasites were lyophilized and extracted with chloroform-methanol (2:1 v/v) to remove lipids and with chloroform-methanol–water (10:10:3 v/v) to remove GPIs. After drying, the residue was resuspended in 100 mM Tris–HCl and 1 mM CaCl_2_ (pH 7.5) and digested with pronase to remove proteins and protein-linked GPIs. The insoluble residue was extracted with 50 mM sodium phosphate (pH 7.2), 4 M guanidine hydrochloride, and 0.5% Triton X-100 and was stirred overnight at 4°C to remove nucleic acids. The residue (insoluble HZ) was recovered by centrifugation, washed three times with double-distilled water and once with 80% 1-propanol, and then dried. The HZ was resuspended in PBS at a final concentration of 10 mg/ml and stored at 4°C.

### Measurement of activated NF-κB by ELISA

The expression of phospho-NF-κB p65 was tested using a sandwich ELISA kit (Cell Signaling, USA) according the manufacturer’s instructions. Each sample was assessed in triplicate. Briefly, total protein was extracted from 2 × 10^6^ cells/well with different treatments. The protein was then added to 96-well plates coated with mouse mAb, and the plates were incubated overnight at 4°C. Next, a phospho-NF-κB p65 rabbit mAb was added, and the horseradish peroxidase (HRP) method was used for detection. Each assay was performed in triplicate.

### Measurement of the histone acetylation levels of the HIV-1 or SIV LTR by ChIP

ChIP analysis was performed according to the online protocol provided by Millipore and procedures described elsewhere [57]. Briefly, cells were cross-linked with 1% formaldehyde for 10 minutes at 37°C; cross-linking was then stopped by the addition of a glycine solution. The cells were washed twice in ice-cold PBS with 1 mM PMSF, resuspended in sodium dodecyl sulfate (SDS) lysis buffer containing 1 mM PMSF and incubated for 10 minutes on ice. The lysates were sonicated to produce DNA fragments with an average length of 500–1000 bp using a Bioruptor D-300 sonicator (Diagenode). The chromatin fragments from J-Lat 10.6 cells (full-length clones) or rhesus macaques’ PBMCs with different treatments were then diluted tenfold with immunoprecipitation (IP) dilution buffer. After pre-clearing with Protein G agarose for 30 minutes at 4°C with agitation, the material was immunoprecipitated with 2 μg anti-AcH3 (catalog no. 17–615, Millipore) or preimmune rabbit IgG (Millipore) via incubation overnight at 4°C with rotation. To collect the immune complexes, appropriate amounts of Protein G agarose were added to each reaction mixture, and the resulting mixtures were rotated for 60 minutes at 4°C. The beads were centrifuged and then sequentially washed for 5 minutes at 4°C with each of the following: low-salt immune-complex wash buffer, high-salt immune-complex wash buffer, LiCl immune-complex wash buffer, and Tris-EDTA buffer. After the immune complexes were eluted by incubation in elution buffer (1% SDS and 0.1 M NaHCO_3_), the supernatants were isolated and further incubated for 4 hours at 65°C with appropriate NaCl concentrations to reverse the cross-linking. Input controls were treated in the same manner. After reverse cross-linking, DNA was purified using an Axygen DNA purification kit (Corning Life Sciences, USA). PCR was performed using HIV-1 or SIV promoter primers spanning the nuc-1 region of the LTR. The primers for the HIV-1 LTR or SIV LTR were as follows: LTR-109 F (5′-TACAAGGGACTTTCCGCTGG-3′) and LTR + 82R (5′-AGCTTTATTGAGGCTTAAGC-3′). The DNA products of ChIP were quantitated by real-time PCR (Bio-Rad CFX96, USA). The phosphorimager data related to the amounts of PCR product obtained from the immunoprecipitated chromatin samples were then compared with the amounts of PCR product obtained for the input DNA. The percent of immunoprecipitated LTR was determined by comparing the cycle threshold values of each reaction with a standard curve generated from the input DNA, and these data are reported as the percent of input.

### HDAC activity assay

An HDAC activity assay was performed using an HDAC Activity Colorimetric Assay Kit (BioVision, Palo Alto, USA) according to the manufacturer’s instructions. Each sample was assessed in triplicate. Briefly, total protein was extracted from 2 × 10^6^ cells/well with different treatments, with a final volume of 200 μl in sterile water. Appropriate volumes of protein solution were then added to 96-well plates containing an appropriate HDAC substrate, HDAC assay buffer and a deacetylated standard; mixed thoroughly; and incubated in 37°C for 1 hour. Finally, lysine developer was added to the wells and incubated in 37°C for 30 minutes to produce a chromophore.

### SIV p27 antigen assay

An SIV p27 antigen assay was performed using an ELISA kit (catalog no. 5436, Advanced Bioscience Laboratories, Kensington, USA) according to the manufacturer’s instructions. Briefly, cell culture supernatant was first treated with phosphate buffer with 2.5% Triton-X-100 to release SIV antigen and then added to 96-well plates coated with a murine mAb to SIV p27. These plates were incubated at 37°C for 1 hour. Next, HRP-labeled mouse mAb to SIV p27 was added and incubated at 37°C for 1 hour, and the HRP method was used for detection. Each assay was performed in triplicate.

### Statistical analysis

Variables in the same phase and group were combined to form a dataset for statistical analysis. Spearman’s correlation analysis was used to analyze the relationship between the parameters tested. The Mann–Whitney U test was used to analyze differences between variables obtained from the *in vivo* tests. Fisher’s exact test was used for comparisons of IUPM under the detection limit, i.e., when the assay could not generate an exact value. One-way ANOVA was used in comparisons of variables for *in vitro* assays. P < 0.05 was considered significant. GraphPad Prism 6 (GraphPad Software) was used for graphing. Statistical analyses were performed using SPSS 16. Empower (R) (www.empowerstats.com, X&Y Solutions, Inc., Boston, MA) and R (http://www.R-project.org) were used for smooth curve fitting.

## Additional files

Additional file 1: Figure S1. Dynamic changes in plasma SIV viral loads and certain immunological parameters in the two groups of monkeys during this study. **(A–C)** Inoculation with SIV resulted in a peak plasma viral load between 10^4^ and 10^7^ copies of viral RNA/ml at week 2, and a viral set point ranging from 10^3^–10^6^ copies of viral RNA/ml of plasma was established at approximately 12–15 weeks. The monkeys showed a decay of viremia to below the limit of detection (100 RNA copies/ml) after approximately 20 weeks of ART. **(A)** The plasma viral load of the monkeys during this study. The line shows the median plasma viral load in both groups during the study. **(B)** The plasma viral load of each monkey in the ART group. **(C)** The plasma viral load of each monkey in the ART + Pc group. The lines in Additional file 1: Figure S1A, B and C show the dynamic changes in the plasma viral load in each monkey during the study. **(D)** The plasma viral load in each phase. Malaria induced a small increase (not significant) in the plasma viral load under ART. The horizontal lines indicate mean values. **(E–F)** Peripheral blood CD4+ T cells in the two groups of monkeys. The number of CD4+ T cells dramatically decreased during acute SIV infection, along with an increase in CD8+ T cells, but both recovered to baseline levels after control of viral replication. **(G–H)** SIV infection induced a degree of immune activation, as represented by an increased proportion of HLA-DR+CD4+ T cells and HLA-DR+CD8+ T cells. The data in Figure S1E–H are shown as the means and SD values. Plasma viral loads from different time points in the same group and phase were combined for statistical analyses. The Mann-Whitney U test was utilized. Additional file 2: Figure S2. The impact of malaria on certain parameters related to activation of the immune system and CD4+ T cells. **(A)** The concentrations of plasma cytokines and activity markers, including IL-2R, IL-7, IL-6 and TNF-α, did not increase during malaria infection under ART. **(B)** The ART + Pc group displayed a significantly higher concentration of plasma neopterin during malaria infection. **(C)** Pc infection induced the expression of CCR5 in CD4+ T cells, which also indicated activation of these cells. **(D)** A significant positive correlation was found between CD4+ T cell activation levels and the plasma neopterin concentration. Parameters from different time points in the same group and phase were combined for statistical analyses. The Mann–Whitney U test was utilized. Spearman’s correlation analysis was used to analyze the relationship. Additional file 3: Figure S3. The dose response to Pf extract in J-Lat cells and monkey resting CD4+ T cells. In total, 2 × 10^6^ cells/well were treated with different concentrations of Pf (Pf 3d7) extract. Each treatment had three replications. Different dose–response patterns were found between J-Lat cells and primary monkey cells. After 48 hours of culture, for J-Lat cells, 5 μg/ml Pf extract could induce more histone acetylation, whereas 50 μg/ml Pf extract could sufficiently induce more histone acetylation in primary monkey cells. One-way ANOVA was used to compare the variables in this figure. Additional file 4: Figure S4. The impact of Pf extract on HDAC activity. Pf extract did not influence the activity of HDAC in J-Lat cells and monkey PBMCs after 24 hours of stimulation. In total, 2 × 10^6^ cells/well were treated with different concentrations of Pf (Pf 3d7) extract. Each treatment had three replications, and each assay had three replications. One-way ANOVA was used to compare the variables in this figure. Additional file 5: Figure S5. The impact of LPS on the histone acetylation level of primary monkey cells. LPS did not influence the histone acetylation level of monkey lymphocytes or CD4+ T cells.

## Abbreviations

HIV-1: Human immunodeficiency virus type-1
SIV: Simian immunodeficiency virus
ART: Antiretroviral therapy
iDNA: Integrated virus DNA
IUPM: Infectious units per million cells
PBMCs: Peripheral blood mononuclear cells
AIDS: Acquired immune deficiency syndrome
T_CM_: Central memory T
T_EM_: Effector memory T
APCs: Antigen-presenting cells
IL-6: Interleukin-6
TNF-α: Tumor necrosis factor-alpha
Pc: *Plasmodium cynomolgi*
IL-7: Interleukin-7
IL-2: Interleukin-2
IFN-γ: Interferon-gamma
PBLs: Peripheral blood lymphocytes
VPA: Valproic acid
PHA: Phytohemagglutinin
MFI: Mean fluorescence intensity
Pf: *Plasmodium falciparum*
Pf dd2: *Plasmodium falciparum* strain dd2
Pf 3d7: *Plasmodium falciparum* strain 3d7
AcH3: Acetylated histone H3
LTRs: Long terminal repeats
Cq: Quantification cycle
HDAC: Histone deacetylase
CHIP: Chromatin immunoprecipitation
*P. knowlesi*: *Plasmodium knowlesi*
*P. coatneyi*: *Plasmodium coatneyi*
HZ: Hemozoin
FACS: Fluorescence-activated cell sorting
PMPA: 9-R-(2-phosphonomethoxypropyl) adenine
FTC: Emtricitabine
RBCs: Red blood cells
