# Supplementary figures and images for: Plasmodium infection reduces the volume of the viral reservoir in SIV-infected rhesus macaques receiving antiretroviral therapy
Source: Retrovirology. 2014 Dec 9;11:112. doi: 10.1186/s12977-014-0112-x (PMC4269176; doi:10.1186/s12977-014-0112-x)

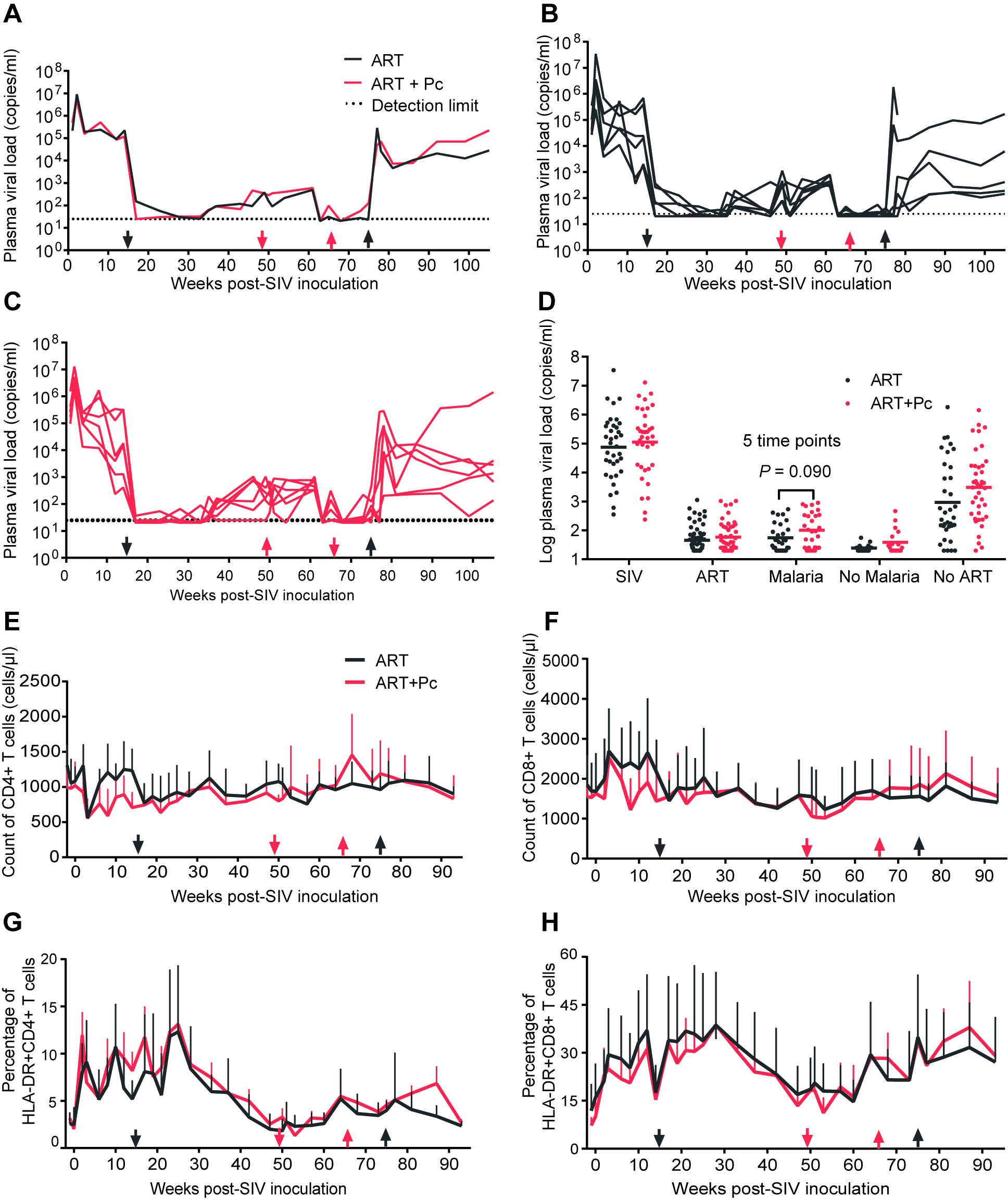

Supplement: Additional file 1: Figure S1. — Dynamic changes in plasma SIV viral loads and certain immunological parameters in the two groups of monkeys during this study. (A–C) Inoculation with SIV resulted in a peak plasma viral load between 104 and 107 copies of viral RNA/ml at week 2, and a viral set point ranging from 103–106 copies of viral RNA/ml of plasma was established at approximately 12–15 weeks. The monkeys showed a decay of viremia to below the limit of detection (100 RNA copies/ml) after approximately 20 weeks of ART. (A) The plasma viral load of the monkeys during this study. The line shows the median plasma viral load in both groups during the study. (B) The plasma viral load of each monkey in the ART group. (C) The plasma viral load of each monkey in the ART + Pc group. The lines in Additional file 1: Figure S1A, B and C show the dynamic changes in the plasma viral load in each monkey during the study. (D) The plasma viral load in each phase. Malaria induced a small increase (not significant) in the plasma viral load under ART. The horizontal lines indicate mean values. (E–F) Peripheral blood CD4+ T cells in the two groups of monkeys. The number of CD4+ T cells dramatically decreased during acute SIV infection, along with an increase in CD8+ T cells, but both recovered to baseline levels after control of viral replication. (G–H) SIV infection induced a degree of immune activation, as represented by an increased proportion of HLA-DR+CD4+ T cells and HLA-DR+CD8+ T cells. The data in Figure S1E–H are shown as the means and SD values. Plasma viral loads from different time points in the same group and phase were combined for statistical analyses. The Mann-Whitney U test was utilized. [file 12977_2014_112_MOESM1_ESM.tiff]

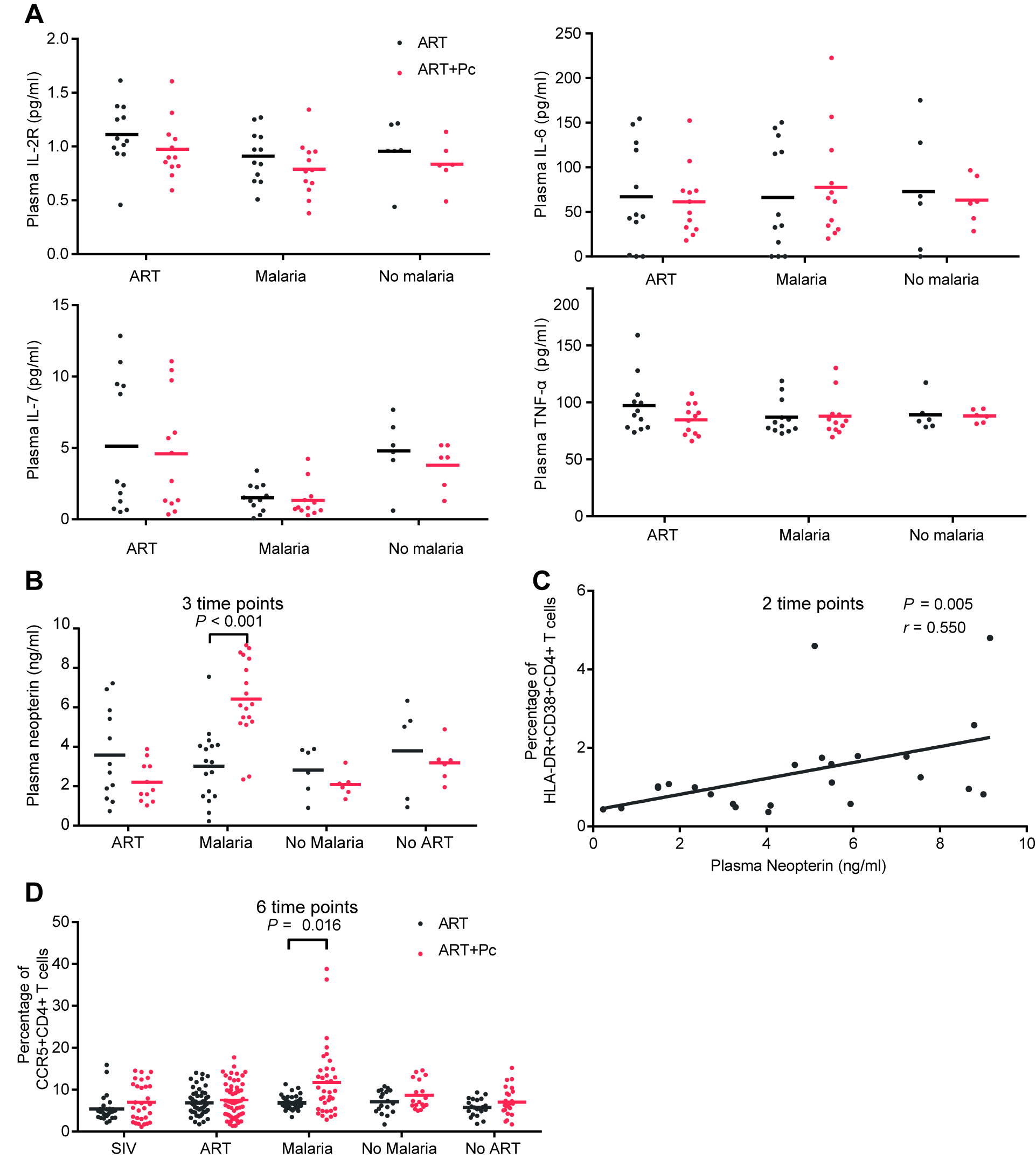

Supplement: Additional file 2: Figure S2. — The impact of malaria on certain parameters related to activation of the immune system and CD4+ T cells. (A) The concentrations of plasma cytokines and activity markers, including IL-2R, IL-7, IL-6 and TNF-α, did not increase during malaria infection under ART. (B) The ART + Pc group displayed a significantly higher concentration of plasma neopterin during malaria infection. (C) Pc infection induced the expression of CCR5 in CD4+ T cells, which also indicated activation of these cells. (D) A significant positive correlation was found between CD4+ T cell activation levels and the plasma neopterin concentration. Parameters from different time points in the same group and phase were combined for statistical analyses. The Mann–Whitney U test was utilized. Spearman’s correlation analysis was used to analyze the relationship. [file 12977_2014_112_MOESM2_ESM.tiff]

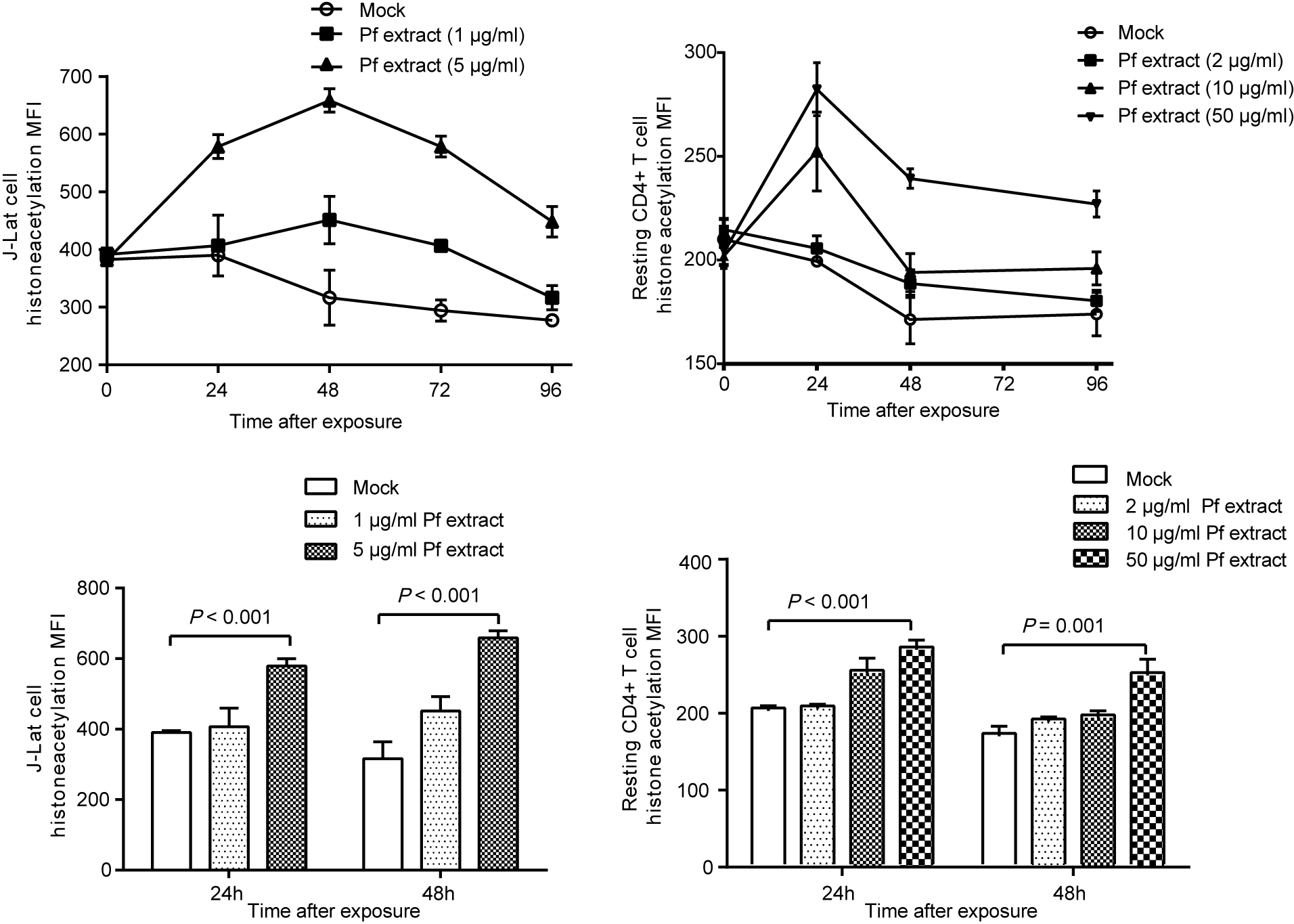

Supplement: Additional file 3: Figure S3. — The dose response to Pf extract in J-Lat cells and monkey resting CD4+ T cells. In total, 2 × 10^6^ cells/well were treated with different concentrations of Pf (Pf 3d7) extract. Each treatment had three replications. Different dose–response patterns were found between J-Lat cells and primary monkey cells. After 48 hours of culture, for J-Lat cells, 5 μg/ml Pf extract could induce more histone acetylation, whereas 50 μg/ml Pf extract could sufficiently induce more histone acetylation in primary monkey cells. One-way ANOVA was used to compare the variables in this figure. [file 12977_2014_112_MOESM3_ESM.tiff]

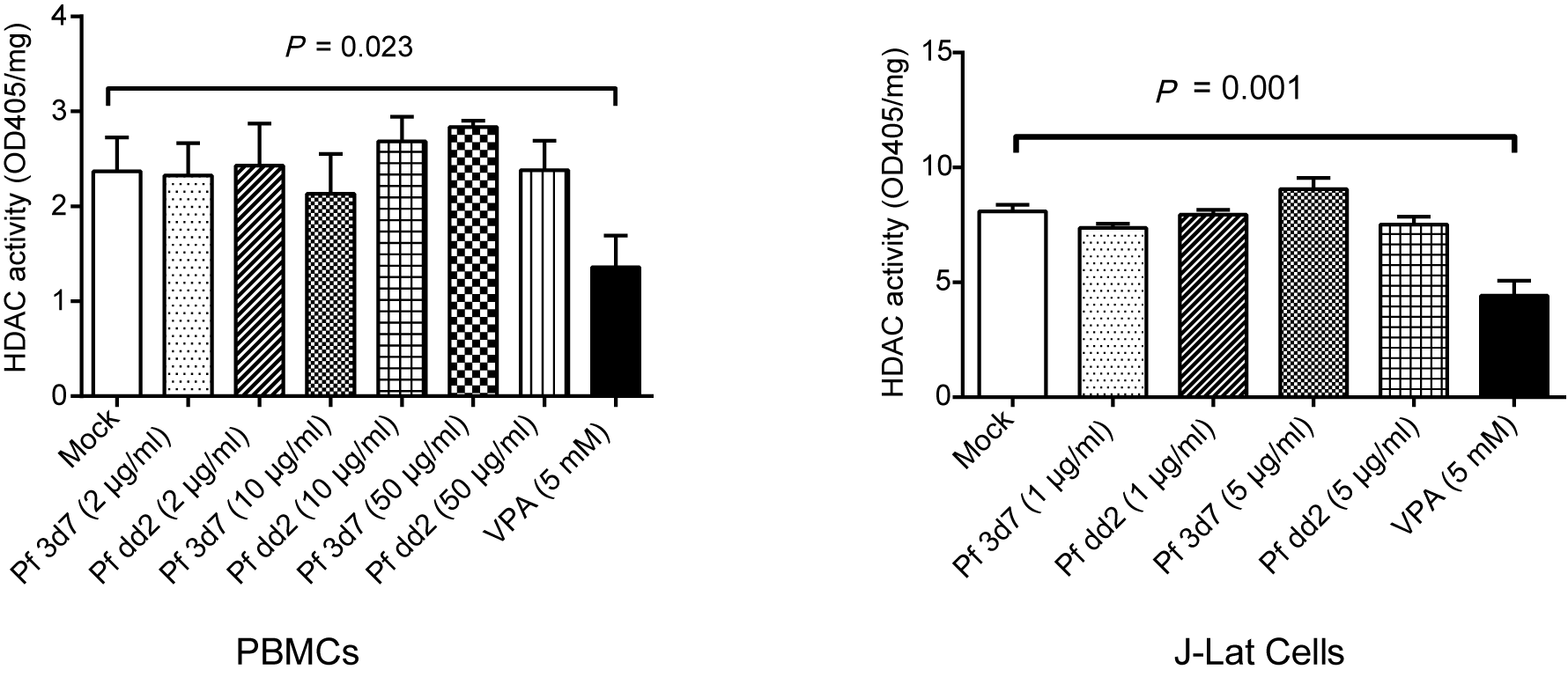

Supplement: Additional file 4: Figure S4. — The impact of Pf extract on HDAC activity. Pf extract did not influence the activity of HDAC in J-Lat cells and monkey PBMCs after 24 hours of stimulation. In total, 2 × 10^6^ cells/well were treated with different concentrations of Pf (Pf 3d7) extract. Each treatment had three replications, and each assay had three replications. One-way ANOVA was used to compare the variables in this figure. [file 12977_2014_112_MOESM4_ESM.tiff]

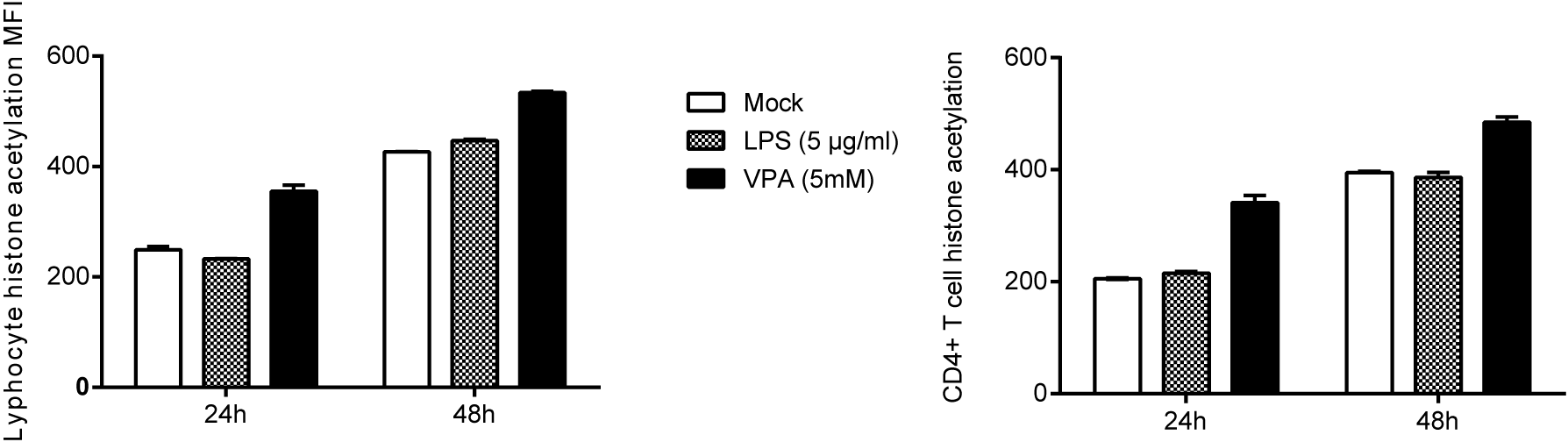

Supplement: Additional file 5: Figure S5. — The impact of LPS on the histone acetylation level of primary monkey cells. LPS did not influence the histone acetylation level of monkey lymphocytes or CD4+ T cells. [file 12977_2014_112_MOESM5_ESM.tiff]
